# Exploring the impact of COVID-19 pandemic on nurses: A cross-sectional study on job burnout and quality of work life

**DOI:** 10.1371/journal.pone.0331247

**Published:** 2025-10-31

**Authors:** Elahe Saleh, Mohamad Ezati Asar, Mohammad Reza Ghaneapur, Nayereh Harati Asl, Marzieh Belji Kangarlou

**Affiliations:** 1 Research Center for Public Health Sciences and Technologies, Semnan University of Medical Sciences, Semnan, Iran; 2 Department of Public Health, Damghan School of Health, Semnan University of Medical Sciences, Semnan, Iran; 3 Department of Occupational Health, Faculty of Medical Sciences, Tehran university of Medical Science, Tehran, Iran; 4 Student´s Scientific Research Center, Tehran Universiry of Medical Sciences, Trehran, Iran; Shahrood University of Medical Sciences, IRAN, ISLAMIC REPUBLIC OF

## Abstract

**Background:**

The COVID-19 pandemic has posed unprecedented challenges for healthcare professionals, especially nurses, affecting their occupational health, burnout levels, and overall quality of working life. Limited research exists in the local context regarding these issues.

**Objectives:**

To evaluate burnout and quality of working life among nurses during COVID-19, and to explore related factors such as exposure level, work shifts, and organizational conditions.

**Methods:**

A cross-sectional study was conducted from January to March 2022 at Velayat Hospital in Damghan, Iran. All 217 active nurses were surveyed using the Maslach Burnout Inventory and Walton’s quality of working life questionnaire. Response rate was 58.5%. Data analysis was performed with SPSS.

**Results:**

The nurses had an average age of 36.3 years; most were female (78.7%) and married (81.8%). The majority held bachelor’s degrees. Regarding quality of working life, 21.3% experienced poor quality, 71.3% moderate, and only 7.4% rated it as good. The lowest scoring domain was “fair and appropriate compensation,” while “development of human capabilities” scored highest. Over 70% experienced moderate to high emotional exhaustion, with higher exposure to COVID-19 correlating with increased burnout. Significant inverse relationships were found between emotional exhaustion and all quality of working life domains. Additionally, the number of shifts was associated with higher levels of depersonalization and perceptions of unfairness, safety, and growth opportunities. Nurses with moderate to severe exposure to COVID-19 reported higher burnout levels. Exposure to COVID-19 also negatively impacted perceptions of organizational legality and social environment.

**Conclusions:**

The COVID-19 crisis has heightened occupational burnout and reduced quality of working life among nurses, risking the quality of healthcare services. Strategies such as workforce reorganization, improved working conditions, and mental health interventions are essential. Policymakers should prioritize supportive measures to enhance resilience and prepare for future crises.

## 1. Introduction

Acute respiratory syndrome caused by the coronavirus disease (COVID-19) was first observed in 2019 in Wuhan, China. This disease rapidly spread around the world, and the World Health Organization declared it a pandemic in February 2020 [1]. Results from studies conducted in workplace environments indicate that 32.60% of workers in various industries reported outcomes related to COVID-19 [2]. Health workers, especially those involved in caring for COVID-19 patients, faced increased risk of contracting this contagious disease due to their position on the front lines of combatting the pandemic, particularly in resource-limited countries [3]. Among them, nurses played a unique role in fighting the COVID-19 pandemic. Nurses risked their lives across various departments, including emergency care, infection control, intensive care, and wards for COVID-19 patients. They showed intense commitment to their profession and to their patients [4]. Exposure to the pathogen, long working hours, psychological pressure, fatigue, burnout, frustration, and physical and psychological violence surrounded healthcare providers [5,6].

Insomnia was cited as a primary factor contributing to the deterioration of mental health among nurses during the COVID-19 pandemic [7], which could impact the quality of work for health workers. Study results showed that throughout the COVID-19 pandemic, nurses faced a wide range of paradoxes, such as distractions from providing care due to a focus on peripheral issues despite empathy and collaboration among nurses; the presence of volunteers amid equipment shortages; insufficient scientific information and unreliable online resources; and work overload in hospitals due to inadequate supplies and the physical avoidance of nurses by the public despite media encouragement for social support [8]. Additionally, the risk of skin disorders, heat stress from prolonged use of personal protective equipment, exposure to toxins from frequent use of disinfectants, psychological distress, chronic fatigue, discrimination, exposure to physical and psychological violence, and even the possibility of harassment were other challenges impacting them [9]. Meanwhile, the findings of studies regarding adherence to COVID-19 protocols in workplace environments, especially in the early months of the COVID-19 pandemic, indicated poor performance [10].

Since the concept of “quality of life” encompasses how an individual evaluates their “well-being” in various aspects of life (through emotional responses to life events, desires, feelings of satisfaction, contentment with life, and satisfaction with work and personal relationships) [11], the quality of working life (QWL) for nurses can be affected under such conditions. QWL is typically assessed in eight domains: fair and adequate compensation, social integrity and cohesion, a safe and healthy working environment, provision of growth opportunities and ongoing security, organizational legality, overall life environment, development of human capabilities, and social dependency of working life [12]. Given the above evidence, occupational burnout among nurses is also noteworthy. Burnout is a type of physical, emotional, and mental exhaustion resulting from prolonged direct exposure to individuals in emotionally stressful conditions. Physical and emotional fatigue due to burnout leads to negative self-perception, negative attitudes toward work, and a lack of connection with others, potentially steering individuals toward various mental and physical health issues [13]. Burnout is one of the common problems among health workers, who are often at greater risk of burnout. For this reason, the term “burnout” is commonly used to describe the syndrome of fatigue in caregiving professions [14]. Despite numerous studies conducted worldwide, the QWL and burnout among nurses during the COVID-19 pandemic have not been evaluated in Iran, and investigations have often been conducted with relatively small sample sizes. Due to the need for comprehensive results and data generalizability, some research has recommended future studies on QWL with larger sample sizes and more precise methodologies [15]. Therefore, the present study was conducted to determine the QWL, burnout among nurses, the dimensions of each, the extent to which these factors influence each other, identify related components during the COVID-19 pandemic, and provide practical solutions in this regard.

## 2. Materials and methods

### 2.1. Research questions

How is the QWL of nurses during the COVID-19 pandemic? What is the level of occupational burnout amongst nurses during the COVID-19 pandemic? What are the factors associated with the QWL and occupational burnout among nurses?

### 2.2. Study design

A cross-sectional assessment study was conducted regarding burnout and the QWL of nurses during the COVID-19 pandemic at Semnan University of Medical Sciences, Iran, from January to March 2022.

### 2.3. Setting

This study was carried out at Valayat Hospital in the city of Damghan, which is affiliated with Semnan University of Medical Sciences, located in northeastern Iran. This hospital is the only hospital in Damghan.

### 2.4. Participants/Inclusion criteria

All 217 nurses working in the clinical departments of Valayat Hospital (the only hospital in Damghan) had an equal chance of participating in this study. The inclusion criteria included being a registered nurse, being employed in the inpatient sections of the hospital, having at least one year of work experience, and willingness to participate in the research.

### 2.5. Data collection

The data collection method was a census. After obtaining the necessary permissions and making multiple separate visits to all clinical departments, the study objectives, implementation process, and method for completing the questionnaires were explained. The questionnaires were distributed to all 217 nurses working in the clinical departments of the hospital.

### 2.7. Instrument with validity and reliability

In this study, a questionnaire consisting of 59 questions in three sections was used:

1. **Demographic and background information** (11 questions).
2. **Walton’s Quality of Work Life Questionnaire** (26 questions).

The Persian Walton’s QWL Scale consists of 26 questions scored on a five-point Likert scale (very low, low, moderate, high, very high). It is categorized into three levels: low (scores 24–56), moderate (57–88), and high (89–120). The total score range for this questionnaire is from 24 to 130. Walton’s QWL includes eight domains: fair and adequate compensation (questions 1–3), social integrity and cohesion (questions 4–6), a safe and healthy working environment (questions 7–9), provision of growth opportunities and ongoing security (questions 10–12), organizational legality (questions 13–15), overall life environment (questions 16–18), development of human capabilities (questions 19–23), and social dependency of working life (questions 24–26). The face and content validity of this questionnaire were reported in the range of 0.85 to 0.90, and its reliability, as measured by Cronbach’s alpha coefficient, was 0.81 [16].

**Definitions of the above dimensions are as follows:**

- **Fair and Adequate Compensation:** The amount employees receive in exchange for their intellectual, physical, or a combination of both types of activities, proportional to social standards, workload, and similar jobs.
- **Social Integrity and Cohesion:** Creating an environment and atmosphere that enhances employees’ sense of belonging to the organization and reinforces their feeling that the organization needs them.
- **Safe and Healthy Work Environment:** Establishing conditions that ensure physical safety at work, along with setting reasonable working hours.
- **Provision of Growth Opportunities and ongoing Security:** Providing opportunities for enhancing individual abilities, career advancement, applying acquired skills, and ensuring income and job security.
- **Social Dependence of Work Life:** Refers to employees’ perceptions of the organization’s social responsibilities.
- **Development of Human Capabilities:** Providing an environment where employees can freely express their opinions without fear of retaliation by higher authorities.
- **Overall Life Balance:** Establishing a balance between work life and other personal responsibilities.
- **Organizational Legality:** Ensuring an environment where employees can freely express their opinions without fear of retaliation by superiors [17].

1. **Maslach Burnout Inventory (22 questions).**

To measure job burnout, Mazlach and Jackson developed a questionnaire in 1986 containing 22 variables across three domains: Emotional Exhaustion (EE) (9 items), Depersonalization (DP) (5 items), and Reduced Personal Accomplishment (PA) (8 items). The Emotional Exhaustion subscale measures feelings of tiredness and low energy, Depersonalization assesses negative responses toward recipients of service, and Personal Accomplishment evaluates feelings of personal inefficacy**.“** Responses were scored on a 7-point Likert scale, with each item scored between 0 and 6 (0 = Never, multiple times = 1; once a month = 2; several times a month = 3; once a week or less = 4; several times a week = 5; daily = 6). The validity and reliability of this questionnaire were assessed and confirmed among Iranian nurses by Moalemi and colleagues [18]. A combination of high scores across the three burnout domains was attributed to a high overall burnout level. Internal consistency for each domain was estimated: Cronbach’s alpha coefficients were 0.84 for EE, 0.76 for DP, 0.79 for PA, and 0.80 for the entire questionnaire. According to the study by Moalemi et al., test-retest reliability coefficients for the three burnout dimensions among Iranian nurses exceeded 0.7 [18].

The items in each domain are as follows:

- Emotional Exhaustion (EE): Items 1, 2, 3, 6, 8, 13, 14, 16, and 20
- Depersonalization (DP): Items 5, 10, 11, 15, and 22
- Reduced Personal Accomplishment (PA): Items 4, 7, 9, 12, 17, 18, 19, and 21

The total score for each domain reflects the individual’s score in that domain.

- For EE, scores above 30 indicate high emotional exhaustion; scores between 18–29 suggest moderate exhaustion; and scores below 17 indicate low emotional exhaustion.
- For DP, scores above 12 reflect high depersonalization; scores between 6–11 indicate moderate depersonalization; and scores below 6 suggest low depersonalization.
- For PA, scores of 40 and above denote low accomplishment; 34–39 moderate; and below 34 high personal accomplishment.

### 2.8. Data collection

The samples were included in the study by census method, therefore, all nurses had the same chance to enter the study. After obtaining the necessary permissions and making repeated and separate visits to all clinical departments, the study objectives, implementation process, and questionnaire completion method were explained. Due to reasons such as: multiplicity and length of work shifts, personal reasons, and in some cases some nurses were infected with covid-19 disease, only 127 questionnaires were completed by nurses and returned to the research team (nurses response rate 58.53%).

### 2.9. Data analysis

The collected data were entered into SPSS-22 software and analyzed and interpreted using descriptive and analytical statistics. A significance level of α ≤ 0.05 was considered for data analysis and interpretation. The normality of the questionnaire scores was assessed using the Shapiro-Wilk test, and based on the obtained results, either the t-test or Mann-Whitney U test was used for comparisons between groups. Finally, the relationship among the different questionnaires was examined using Spearman’s correlation coefficients. To determine the impact of burnout on the QWL among nurses in the presence of other variables, a linear regression model was used, with the stepwise regression method employed for variable entry into the model.

The collected data were entered into SPSS-22 software and analyzed and interpreted using descriptive and analytical statistics. A significance level of α ≤ 0.05 was considered for data analysis and interpretation. The normality of the questionnaire scores was assessed using the Shapiro-Wilk test, and based on the obtained results, either the t-test or Mann-Whitney U test was used for comparisons between groups. Finally, the relationship among the different questionnaires was examined using Spearman’s correlation coefficients. To determine the impact of burnout on the QWL among nurses in the presence of other variables, a linear regression model was used, with the stepwise regression method employed for variable entry into the model.

### 2.10. Ethical considerations

This study was conducted in accordance with the ethical standards and regulations as approved by the Research Ethics Review Committee of Semnan University of Medical Sciences (Approval No: IR.SEMUMS.REC.1400.206, Code No: A-10-90-3, approval date: 2022-1-1). The purpose of the research and the method of completing the questionnaires were explained to nurses and informed written consent was obtained from all nurses for participation in the study. Moreover, before collecting data participants were ensured of the confidentiality of their personal data and the relevant ethical aspects. In all cases where the questioner referred for training and data collection, personal protection protocols regarding the prevention of contamination and the spread of the disease COVID-19 (notified by the Ministry of Health of the Islamic Republic of Iran) were considered and implemented by the researchers and participants.

## 3. Results

### 3.1. Characteristics of the sample

Of the 127 nurses, 100 (78.7%) were female and 27 (21.3%) were male. The means and standard deviations of the nurses’ age were 36.26 ± 8 and work experience was 12.97 ± 8.37. Most of the nurses (81.8%) were married, 34.2% of married nurses had no children, 52% had one to two children, and 13.8% had three or more children. Nintey-two point nine (92.9%) of the participants held a bachelor’s degree, 5.5% had a master’s degree, and the remainder had an associate degree. Only 24.8% of the nurses had the mandatory number of monthly shifts.. Thirty-Three point six (33.6%) of the nurses worked in non-admitted care departments (dialysis, outpatient clinics, nursing offices, emergency), 40.3% in general wards (internal medicine, surgery, obstetrics, pediatrics), and 26.1% in special care units (ICU, CCU, operating rooms). Regarding exposure to COVID-19 patients, 19.6% of the nurses had low exposure, 22.5% had moderate exposure, and 57.8% had high exposure.

### 3.2. Status of the quality of working life

**Fig 1:**
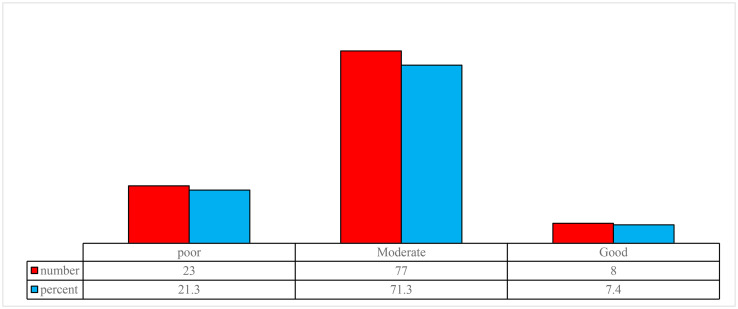
Number and percentage of nurses surveyed by quality of work life status.

The mean and standard deviation of the overall QWL score for nurses, based on the eight dimensions of the Walton questionnaire, was 67.32 ± 14.29. Based on this, 23 nurses (21.3%) had poor work-life quality, 77 nurses (71.3%) had moderate work-life quality, and only 8 nurses (7.4%) exhibited good work-life quality (Fig 1). Higher scores in each dimension of work-life quality indicate greater desirability of work quality in that dimension. Considering that the calculated means in most dimensions of nurses’ work-life quality are below the median of the range, it can be concluded that, in most of the examined dimensions, nurses’ work-life quality is at a moderate to low level. The lowest level of work-life quality among the nurses, based on mean scores, was in the dimension “**Fair and Adequate Compensation”** (6.21 ± 2.36), while the highest was in **“Development of Human Capabilities”** (12.21 ± 2.68) (Table 1).

**Table 1:** Mean and standard deviation of QWL of study participants.

Variables	FAC	SHWE	DHC	GOOS	SIC	RLO	OLA	SDWL	Total
	Mean (SD)	Mean (SD)	Mean (SD)	Mean (SD)	Mean (SD)	Mean (SD)	Mean (SD)	Mean (SD)	Mean (SD)
	6.21 (2.36)	8.10 (1.89)	12.21 (2.68)	10.16 (2.81)	7.98 (2.29)	7.57 (2.1)	6.93 (2.05)	8.92 (2.42)	67.32 (14.29)
Range of scores	3-15	3-15	5-25	3-15	3-15	3-15	3-15	3-15	26-130

FAC: Fair and Adequate Compensation, SHWE: Safe and Healthy Work Environment, DHC: Development of Human Capabilities, GOOS: Growth Opportunities and Ongoing Security, SIC: Social Integrity and Cohesion, RLO: Rule of Law within the Organization, OLS: overall life atmosphere, SDWL: social dependency of work life.

### 3.3. Components related to the quality of working life

In this study, significant relationships were found between the number of shifts and fair compensation (p = 0.004), safe and healthy working conditions (p = 0.023), and provision of growth opportunities and security (p = 0.042). Significant relationships were found for all QWL dimensions based on employment status. There was a significant correlation between the years of activity and integration and cohesion in the organization (p = 0.018) as well as rule of law in the organization (p = 0.034). A significant relationship was found between the level of exposure to COVID-19 patients and the provision of growth opportunities and security (p = 0.020), rule of law in the organization (p = 0.020), overall life atmosphere (p = 0.032), and social dependency of work life (p = 0.013) (Table 2).

**Table 2:** Mean and standard deviation of QWL of study participants based on sociodemographic characteristics and selected variables.

Variables	Subscales	FAC Mean (SD)	SHWE Mean (SD)	DHC Mean (SD)	GOOS Mean (SD)	SIC Mean (SD)	RLO Mean (SD)	OLA Mean (SD)	SDWL Mean (SD)	Total
**Sex**	Female	6.08 (2.3)	8.12 (1.9)	12.19 (2.6)	10.01 (2.8)	7.9 4 (2.2)	7.44 (1.9)	6.92 (2.0)	8.77 (2.4)	
	Male	6.69 (2.3)	8.00 (1.8)	12.27 (2.9)	10.70 (2.7)	8.15 (2.6)	8.04 (2.5)	6.95 (2.0)	9.5 (2.4)	
	***p-value***	0.229	0.735	0.560	0.166	0.752	0.423	0.794	0.397	0.900
**MS**	Single	6.74 (2.1)	8.04 (1.5)	11.96 (1.8)	10.08 (2.2)	7.42 (1.9)	7.54 (1.8)	6.91 (1.7)	8.96 (2.0)	
	Married	6.09 (2.4)	8.11 (1.9)	12.26 (2.8)	10.18 (2.9)	8.12 (2.3)	7.57 (2.1)	6.94 (2.1)	8.91 (2.5)	
	***p-value***	0.201	0.840	0.460	0.850	0.120	0.960	0.990	0.870	0.970
**NC**	Non	6.19 (2.2)	8.02 (1.6)	11.93 (2.7)	9.68 (2.4)	7.64 (2.3)	7.26 (2.2)	6.72 (2.3)	8.73 (2.2)	
	1-2	6.17 (2.4)	8.10 (1.9)	12.47 (2.6)	10.39 (2.9)	8.14 (2.2)	7.64 (2.0)	6.98 (1.9)	8.87 (2.5)	
	> 2	6.23 (2.3)	8.18 (2.3)	12.06 (3.3)	10.56 (2.9)	8.37 (2.2)	7.94 (2.1)	7.40 (1.8)	9.65 (2.4)	
	***p-value***	0.960	0.910	0.570	0.240	0.250	0.600	0.568	0.230	0.630
**NS**	Standard shifts	7.13 (2.3)	8.59 (1.6)	12.37 (1.9)	10.93 (2.4)	8.20 (2.0)	7.73 (1.7)	7.40 (1.4)	9.03 (1.6)	
	Exceeding shifts	5.70 (2.1)	7.98 (1.9)	12.12 (2.9)	9.82 (2.8)	7.95 (2.4)	7.50 (2.2)	6.82 (2.2)	8.88 (2.7)	
	***p-value***	0.004*	0.023*	0.900	0.042*	0.650	0.600	0.320	0.890	0.140
**WE**	< 5	5.70 (2.5)	8.32 (1.8)	12.08 (3.0)	9.58 (2.8)	7.72 (2.6)	7.36 (2.5)	6.42 (2.5)	8.71 (2.3)	
	[5-13)	5.11 (2.0)	7.11 (2.0)	9.11 (3.2)	9.11 (3.2)	8.22 (2.3)	7.33 (1.9)	6.80 (2.2)	8.74 (2.4)	
	[13-19)	5.83 (2.3)	7.83 (1.8)	10.00 (3.0)	10.00 (3.0)	7.13 (2.0)	6.96 (2.1)	6.78 (2.4)	8.56 (2.4)	
	> 19	6.18 (2.1)	8.67 (2.2)	10.95 (2.1)	10.95 (2.1)	9.24 (2.2)	8.77 (2.0)	7.88 (1.1)	9.91 (2.6)	
	***p-value***	0.473	0.064	0.164	0.094	0.018*	0.034*	0.145	0.320	< 0.001*
**ES**	job security	3.00 (0)	4.87 (1.6)	6.62 (1.6)	5.50 (2.2)	5.86 (1.2)	4.37 (1.4)	4.62 (3.1)	4.87 (2.7)	
	lack of job security	6.42 (2.3)	8.32 (1.7)	12.59 (2.2)	10.49 (2.5)	8.11 (2.2)	7.78 (1.9)	7.10 (1.8)	9.20 (2.1)	
	***p-value***	< 0.001*	< 0.001*	<0.001*	< 0.001*	0.005*	< 0.001*	0.007*	< 0.001*	0.100
**LA**	Non-inpatient	5.92 (2.2)	7.77 (1.7)	12.27 (2.1)	10.59 (2.7)	7.78 (2.3)	7.37 (2.4)	6.77 (1.9)	9.27 (2.6)	
	General	6.41 (2.3)	8.00 (1.9)	11.98 (2.4)	8.02 (2.1)	8.02 (2.1)	7.60 (1.8)	7.11 (2.0)	8.60 (2.5)	
	Special care	6.06 (2.6)	8.55 (2.1)	12.29 (2.8)	8.03 (2.6)	8.03 (2.6)	7.48 (2.0)	6.75 (2.3)	8.60 (2.0)	
		0.610	0.290	0.830	0.900	0.900	0.860	0.520	0.400	0.560
**LECP**	Low	6.00 (2.2)	7.53 (2.3)	12.00 (3.1)	8.95 (2.6)	7.68 (2.4)	6.95 (1.7)	6.79 (1.8)	8.05 (2.7)	
	Moderate	5.09 (2.1)	7.83 (2.0)	11.26 (2.5)	8.83 (3.0)	7.22 (2.3)	6.56 (2.2)	5.86 (2.4)	7.91 (2.3)	
	High	5.67 (2.0)	8.15 (1.8)	12.72 (2.8)	10.45 (2.7)	8.47 (2.4)	8.03 (2.2)	7.38 (2.1)	9.59 (2.5)	
		0.300	0.790	0.080	0.020*	0.070	0.020*	0.032*	0.013*	0.052

*p-value<0.05, MS: Marital Status, NC: Number of Children, NS: Number of Shifts, WE: Worke Experience, ES: Employment Status, LA: Location of Activity, LECP: Level of Exposure to COVID-19 Patient, FAC: Fair and Adequate Compensation, SHWE: Safe and Healthy Work Environment, DHC: Development of Human Capabilities, GOOS: Growth Opportunities and Ongoing Security, SIC: Social Integrity and Cohesion, RLO: Rule of Law within the Organization, OLS: overall life atmosphere, SDWL: social dependency of work life.

### 3.4. Burnout status

In this study, nearly 70% of the participants experienced medium or high frequencies of emotional fatigue, and more than 65% described the intensity of this fatigue as medium or high. Regarding depersonalization, 57% of the participants reported it occurring at a low frequency, while the rest experienced it at a medium or high frequency. The intensity of depersonalization was mostly low (62.5%). Only 12.9% of individuals reported low frequencies of failure, whereas 60.4% experienced high frequencies of failure, and 60.8% rated the intensity of their failures as high. (Table 3).

**Table 3:** Frequency (Percentage) of participants based on the frequency and intensity of job burnout.

	Low				Moderate				High			
	Frequency		Intensity		Frequency		Intensity		Frequency		Intensity	
**Variables**	**N**	**%**	**N**	**%**	**N**	**%**	**N**	**%**	**N**	**%**	**N**	**%**
**EE**	31	30.1	36	34.6	43	41.7	60	57.7	29	28.2	8	7.7
**DP**	61	57	60	62.5	33	30.8	35	36.5	13	12.2	1	1
**PA**	13	12.9	15	14.7	27	26.7	25	24.5	61	60.4	62	60.8

EE: Emotional Exhaustion, DP: Depersonalization, PA: Personal Accomplishment

### 3.5. Components related to burnout

In this study, a significant relationship was found between the frequency of emotional exhaustion and employment status (p-value = 0.02). Additionally, individuals with low exposure to COVID-19 patients experienced significantly lower levels of intensity of emotional exhaustion (p-value = 0.02) and frequency of emotional exhaustion (p-value = 0.08) compared to those with moderate or higher exposure. The intensity of depersonalization was significantly related to the number of children (p-value = 0.047). A significant relationship was found between the number of shifts and the intensity of depersonalization (p = 0.012) as well as the reduced personal accomplishment (p = 0.046) (Table 4).A direct and significant relationship was found among all components of quality of working life.The frequency of emotional exhaustion was negatively and significantly related to all dimensions of work-related quality of life except for Fair and Adequate Compensation. A significant inverse relationship was found between the intensity of emotional exhaustion and all other dimensions of work-related quality of life.A significant relationship was found between the frequency of depersonalization and all dimensions of work-related quality of life except for Opportunities for Growth and Security and Fair Compensation. The intensity of depersonalization, except for the dimensions of Organizational Legality and Fair Compensation, had a significant relationship with other dimensions of work-related quality of life. The dimensions of Opportunities for Growth and Security and Fair and Adequate Compensation showed no significant relationship with the Personal Accomplishment. the other dimensions showed significant relationships.The intensity of the Personal Accomplishment was significantly correlated with Fair and Adequate Compensation, Development of Human Capabilities, and Overall Life Balance.There was a significant relationship among all dimensions of burnout. There was a direct relationship between depersonalization and emotional exhaustion, and both dimensions had an inverse relationship with the Personal Accomplishment (Table 5).

**Table 4:** Mean and standard deviation of job burnout among study participants based on socio-demographic characteristics and selected variables.

Variables		EE		DP		PA	
		Frequency	Intensity	Frequency	Intensity	Frequency	Intensity
		Mean (SD)	Mean (SD)	Mean (SD)	Mean (SD)	Mean (SD)	Mean (SD)
**Sex**	Female	24.01 (10.25)	27.26 (10.40)	5.22 (5.03)	6.24 (6.00)	31.45 (7.35)	34.78 (7.64)
	Male	23.09 (8.49)	26.62 (7.72)	6.24 (5.49)	7.46 (4.96)	30.35 (8.59)	32.91 (10.66)
	***p-value***	0.69	0.89	0.35	0.14	0.54	0.35
**MS**	Single	25.19 (8.42)	27.76 (7.87)	7.22 (5.02)	7.23 (5.03)	30.64 (8.13)	33.82 (10.24)
	Married	23.45 (10.21)	26.95 (10.29)	5.20 (5.16)	6.32 (5.97)	31.35 (7.51)	34.51 (7.88)
	***p-value***	0.23	0.68	0.17	0.24	0.70	0.77
**NC**	Non	24.05 (10.75)	27.36 (10.75)	6.79 (5.23)	8.27 (6.31)	29.7 (8.84)	32.78 (9.89)
	1-2	23.58 (10.49)	26.75 (10.58)	4.88 (5.52)	5.62 (5.9)	32.02 (6.70)	35.25 (7.45)
	> 2	23.42 (4.40)	26.80 (5.48)	4.31 (2.87)	5.33 (3.62)	33.17 (7.06)	35.43 (6.62)
	***p-value***	0.93	0.97	0.051	0.047	0.24	0.36
**NS**	Standard shifts	23.00 (9.14)	25.16 (10.72)	4.16 (3.90)	4.28 (4.33)	29.91 (6.17)	32.2 (7.25)
	Exceeding shifts	24.19 (10.24)	28.04 (9.50)	5.91 (5.53)	7.38 (6.14)	32.26 (7.90)	36.00 (8.33)
	***p-value***	0.97	0.55	0.15	0.012*	0.20	0.046*
**WE**	< 5	23.29 (12.59)	27.55 (13.25)	5.64 (5.52)	7.60 (7.36)	31.86 (9.26)	36.86 (9.11)
	5-13	23.86 (8.89)	27.67 (8.82)	5.69 (5.83)	7.59 (6.64)	32.00 (6.27)	37.05 (6.63)
	13-19	22.84 (9.06)	28.79 (8.51)	4.74 (6.17)	5.29 (6.46)	32.17 (6.21)	35.17 (5.75)
	> 19	24.25 (10.85)	25.89 (10.21)	4.59 (3.24)	5.72 (3.27)	36.20 (7.23)	38.44 (7.74)
	***p-value***	0.98	0.69	0.5	0.7	0.29	0.65
**ES**	job security	35.40 (9.76)	34.80 (11.3)	10.00 (9.08)	10.60 (8.76)	28.60 (10.11)	32.40 (9.63)
	lack of job security	23.21 (9.53)	26.73 (9.64)	5.23 (4.80)	6.31 (5.57)	31.33 (7.51)	34.46 (8.37)
	***p-value***	0.02*	0.21	0.2	0.18	0.44	0.59
**LA**	Non-inpatient	25.73 (10.35)	27.90 (9.02)	7.41 (6.60)	7.77 (5.83)	32.19 (7.52)	34.27 (8.47)
	General	22.62 (8.30)	25.24 (7.76)	4.16 (4.12)	5.22 (4.67)	32.36 (7.64)	34.70 (8.23)
	Special care	23.43 (12.34)	28.86 (13.71)	5.22 (4.45)	7.59 (7.09)	28.81 (7.18)	34.07 (8.26)
		0.35	0.34	0.09	0.16	0.13	0.95
**LECP**	Low	18.31 (9.45)	22.00 (9.23)	3.12 (3.04)	3.81 (3.43)	31.78 (9.21)	37.06 (9.75)
	Moderate	26.94 (10.56)	32.56 (11.12)	6.23 (4.41)	8.17 (6.3)	30.82 (6.63)	33.88 (7.14)
	High	23.11 (11.05)	26.31 (10.35)	6.02 (6.34)	7.40 (6.77)	33.40 (7.89)	37.29 (7.83)
		0.08	0.02*	0.10	0.09	0.48	0.33

*p-value<0.05, MS: Marital Status, NC: Number of Children, NS: Number of Shift, WE: Worke Experience, ES: Employment Status, LA: Location of Activity, LECP: Level of Exposure to COVID-19 Patient.

*p-value<0.05, MS: Marital Status, NC: Number of Children, NS: Number of Shift, WE: Worke Experience, ES: Employment Status, LA: Location of Activity, LECP: Level of Exposure to COVID-19 Patient, FAC: Fair and Adequate Compensation, SHWE: Safe and Healthy Work Environment, DHC: Development of Human Capabilities, GOOS: Growth Opportunities and Ongoing Security, SIC: Social Integrity and Cohesion, RLO: Rule of Law within the Organization, OLS: overall life atmosphere, SDWL: social dependency of work life, EE: Emotional Exhaustion, DP: Depersonalization, PA: Personal Accomplishment, F: Frequency, I: Intensity.

**Table 5:** Relationship between components of job quality of life and job burnout.

Variables			FAC	SHWE	DHC	GOOS	SIC	RLO	OLA	SDWL	EE		DP		PA	
											F	I	F	I	F	I
**FAC**		***r***	1													
		***p***	–													
**SHWE**		***r***	0.51*	1												
		***p***	<0.001	–												
**DHC**		***r***	0.35*	0.58*	1											
		***p***	<0.001	<0.001	–											
**GOOS**		***r***	0.58*	0.49*	0.58*	1										
		***p***	<0.001	<0.001	<0.001	–										
**SIC**		***r***	0.38*	0.44*	0.56*	0.45*	1									
		***p***	<0.001	<0.001	<0.001	<0.001	–									
**RLO**		***r***	0.49*	0.57*	0.65*	0.58*	0.63*	1								
		***p***	<0.001	<0.001	<0.001	<0.001	<0.001	–								
**OLA**		***r***	0.39*	0.52*	0.47*	0.41*	0.58*	0.56*	1							
		***p***	<0.001	<0.001	<0.001	<0.001	<0.001	<0.001	–							
**SDWL**		***r***	0.42*	0.49*	0.62*	0.48*	0.43*	0.61*	0.53*	1						
		***p***	<0.001	<0.001	<0.001	<0.001	<0.001	<0.001	<0.001	–						
**EE**	**F**	***r***	−0.16	−0.50*	−0.47*	−0.23*	−0.29*	−0.35*	−0.36*	−0.3*	1					
		***p***	0.1	<0.001	<0.001	0.022	0.003	<0.001	<0.001	0.002	–					
	**I**	***r***	−0.25*	−0.50*	−0.50*	−0.29*	−0.37*	−0.33*	−0.41*	−0.31*	0.88*	1				
		***p***	0.012	<0.001	<0.001	0.003	<0.001	<0.001	<0.001	<0.001	<0.001	–				
**DP**	**F**	***r***	−0.08	−0.24*	−0.39*	−0.09*	−0.23*	−0.2*	−0.26*	0.27*	0.53*	0.42*	1			
		***p***	0.417	0.01	<0.001	0.34	0.015	0.04	0.001	0.006	<0.001	<0.001	–			
	**I**	***r***	−0.18	−0.3*	−0.40*	−0.18	−0.19	−0.18	−0.26*	−0.25*	0.49*	0.42*	0.92*	1		
		***p***	0.071	0.002	<0.001	0.08	0.061	0.064	0.011	0.012	<0.001	<0.001	<0.001	–		
**PA**	**F**	***r***	−0.1	0.22*	0.34*	0.03	0.3*	0.26*	0.46*	0.25*	−0.30*	−0.32*	−0.33*	−0.36*	1	
		***p***	0.33	0.03	0.001	0.8	0.003	0.01	<0.001	0.011	0.002	0.002	0.001	<0.001		
	**I**	***r***	−0.3*	0.15	0.34*	−0.10	0.15	0.16	0.31*	0.18	−0.32*	−0.21*	−0.38*	−0.34*	0.79*	1
		***p***	0.003	0.12	<0.001	0.31	0.15	0.101	0.002	0.067	0.001	0.037	<0.001	<0.001	<0.001	–

The results obtained from the regression model regarding the impact of burnout on quality of working life, considering other variables, indicated that the most significant factors affecting the QWL of nurses are “the intensity of emotional exhaustion” and “their employment status” (Table 6).

**Table 6:** Regression model of burnout on quality of working life.

Variables	Regression coefficient (beta)		Standard Deviation	t-statistic	p-value
	Unstandardized	Standardized			
**Constant**	48.05		15.22	3.16	0.003
**Intensity of emotional exhaustion**	−0.69	−0.46	0.18	−3.91	<0.001
**Type of employment**					
**Job security**					
**lack of job security**	**20.18**	**0.36**	**6.61**	**3.05**	**0.004**

*Variables included in the model: gender, marital status, number of children, number of shifts, type of employment, years of service, work location, level of exposure to COVID-19 patients, intensity of depersonalization, intensity of feelings of ineffectiveness, intensity of emotional exhaustion.

The remaining variables in the model, using the stepwise regression method, included: intensity of emotional exhaustion and type of employment.

## 4. Discussion

Out of 217 questionnaires distributed among nurses, only 127 were completed and returned (for a response rate of 58.53%). Reasons cited included the high number of work shifts, personal issues, and nurses contracting COVID-19. Regarding exposure to patients with COVID-19, only 19.6% of nurses had low exposure. The results of this study largely aligned with the findings of Mokhtari et al., where only 20% of nurses were reported to have very low exposure [19]. In this study, 7.4% of nurses were rated to have good quality of working life, while the rest were rated at a weak to moderate quality of working life. The findings of this research are largely consistent with those of Mohammadi et al., which reported that 4.9% of nurses had good quality of life. After determining and comparing the eight domains of quality of working life, it was found that the lowest average scores were associated with “fair and adequate compensation,” while the highest scores pertained to the domain of “development of human capabilities.” This finding contrasts with the results of Mohammadi et al., who identified “social integration and cohesion” and “safe and healthy work environment” as having the lowest and highest scores among the dimensions of QWL for nurses. This discrepancy likely indicates that other individual, organizational, and social factors may impact the quality of nurses’ work life. Among the eight domains of nurses’ quality of working life, the lowest average score was related to “fair and adequate compensation,” while the highest average score was related to the domain of “development of human capabilities.” These results differ from those of Mohammadi et al., who reported “social integration and cohesion” and “safe and healthy work environment” as having the lowest and highest average quality scores for nurses [16].

In the current study, no significant relationship was observed between “marital status” and “number of children” and the QWL of nurses. In the study conducted by Mohamadzadeh Tabrizi et al., no particular correlation was noted between marital status and the QWL of nurses during the COVID-19 pandemic. Therefore, the results are consistent with those of the mentioned study [20]. It is noteworthy that in their study, the number of children was not examined, and the SF-36 questionnaire was used to assess quality of life, whereas the present study employed a QWL questionnaire that largely addresses factors related to the workplace. Additionally, Mohamadzadeh Tabrizi et al. established a significant relationship between physical activity and smoking among nurses and their quality of life during the pandemic; however, these two components were not considered in the present study [20].

More than 70% of participants in this study experienced emotional exhaustion, and 68% of them described the intensity of this exhaustion as “moderate or high.” No significant relationship was found between the level of emotional exhaustion of nurses and their “gender” and “years of work.” These results were inconsistent with the findings from a similar study conducted by Murat et al., which showed that male nurses and those with a bachelor’s degree who had 1–10 years of experience experienced greater emotional exhaustion. The findings of the current research indicated that the “intensity of emotional exhaustion” among individuals with “low exposure to COVID-19 patients” was significantly lower than among those with “moderate or high exposure.” However, no related studies could be found on this specific topic. The study by Murat et al. demonstrated that the “positive COVID-19 test” among nurses who “were not willing to work voluntary shifts” was significantly higher than among others [21]. Although a significant statistical relationship was observed between the “number of work shifts” of nurses and their “intensity of depersonalization” and “intensity of feelings of ineffectiveness,” the aspect of “voluntary work shifts” was not addressed by the researchers.

Based on the findings, a significant inverse relationship existed between “the intensity of emotional exhaustion” and “all dimensions of quality of life.” In this study, except for the dimension of “fair and adequate compensation,” a significant inverse relationship was observed between all “dimensions of working life quality” and the “frequency of emotional exhaustion” among nurses. Aside from “fair and adequate compensation,” significant inverse relationships were found between “depersonalization (both frequency and intensity)” and all dimensions of nurses’ quality of working life. “Frequency of feelings of ineffectiveness” had no significant relationship with the dimensions of “fair and adequate compensation” and “provision of growth opportunities and security,” but had a direct and significant relationship with other dimensions of quality of working life. Additionally, “intensity of feelings of ineffectiveness” had a direct and significant relationship only with the dimensions of “fair and adequate compensation,” “overall life environment,” and “development of human capabilities” of nurses’ quality of working life.

In the current study, a significant relationship was found between job stability, or “employment status,” and “all dimensions of nurses’ quality of life,” but no similar study was identified in this regard. According to the findings of the research, a significant relationship existed between “all dimensions of burnout.” No similar research was found regarding this. A direct and significant relationship was observed between “depersonalization” and “emotional exhaustion,” while these two dimensions had an inverse relationship with “feelings of ineffectiveness.” The study found that there was a significant statistical relationship between “number of shifts” and “fair compensation,” “safe and healthy working conditions,” and “provision of growth opportunities and security.” This study demonstrated that there is a significant relationship between “level of exposure to COVID-19 patients” and “provision of growth opportunities and security,” “organizational legality,” “overall life environment,” and “social dependency of working life.” This issue not only impacts the personal aspects of nurses’ lives but can also influence the managerial aspects governing individual working conditions and the resulting consequences. The findings of the present research support the reality that the quality of life for nurses has decreased during the COVID-19 pandemic, which aligns with findings from other studies in this regard. The researchers believe that this situation may occur in similar conditions, thus highlighting the urgent necessity for prevention and treatment of mental health disorder symptoms among nurses (and healthcare providers) [22].

The high prevalence of depression and anxiety among nurses during the COVID-19 pandemic and its association with psychosocial factors [23] has coincided with a decrease in job satisfaction among nurses, emergence of psychological distress, and a tendency to leave their jobs [24]. The aforementioned factors have affected nurses’ perceptions of their QWL [25], and insufficient occupational health and safety measures have created conditions for an increase in work-related illnesses, high absenteeism rates, reduced productivity and efficiency, and consequently decreased quality of nursing care [26].

## 5. Conclusion

The establishment of a significant relationship between “all dimensions of burnout” reveals that placing nurses in similar conditions may not only impact the personal aspects of their lives but could also have managerial implications for individual working conditions and the resulting consequences. Research findings during the COVID-19 pandemic also indicated that, compared to before, a greater number of nurses left their jobs, underscoring that resilience affects nurses’ job satisfaction and consequently impacts the quality of care as well. Therefore, strong resilience can enhance the provision of high-quality nursing care by nurses in such situations and improve their job satisfaction [27]. The findings of this study and the supporting documents from similar studies demonstrate that in crises such as the COVID-19 pandemic, the quality of services provided in the healthcare system is severely compromised, leading to irreparable harm to the human and social capital of the organization—its workforce. Thus, reorienting human resources and reviewing services and arrangements to prepare for similar crises is recommended. Given the scale of the problem and the need to determine the effects of COVID-19 on human health, violence, and other consequences, researchers must mobilize to address these issues [28]. This emerging occupational challenge emphasizes the need for novel strategies and intervention procedures to prevent burnout and secondary trauma, aimed at reducing the risks of adverse mental health outcomes in similar situations among healthcare defenders [29], which should be considered by decision-makers and planners in the healthcare system.

### Limitations of the study

1. **Limited Sampling:** The study was conducted only among nurses in a specific region, which may limit the generalizability of the findings to other areas.
2. **Duration of the Study:** Conducting the research over a fixed period may not capture changes in the QWL of nurses over time, possibly leading to results influenced by specific temporal conditions.
3. **Use of Questionnaires:** The data collection instrument relied on self-reported questionnaires, which may introduce subjectivity and variability in responses due to personal interpretation.
4. **Exclusion of External Factors:** Environmental and social variables that could impact working life quality and burnout were not comprehensively addressed in this study.
5. **Lack of Diversity in Variables:** Important factors such as the economic and social status of nurses and their effects on quality of life and burnout were not included in the analysis.
6. **Cross-Sectional Nature:** The study employed a cross-sectional design, limiting its ability to identify long-term changes in working life quality and occupational burnout.
7. **Failure to Examine Individual Behaviors:** Key variables such as lifestyle choices and health-related behaviors, which might influence working life quality, were not thoroughly investigated.

These limitations highlight areas for future research and can assist scholars in addressing existing weaknesses in subsequent studies.

## References

[1] Organization WH. WHO Director-General’s remarks at the media briefing on 2019-nCoV on 11 February 2020. 2020.

[2] Malekpour F, Ebrahimi H, Yarahmadi R, Mohammadin Y, Kharghani Moghadam SM, Soltanpour Z. Prevention measures and risk factors for COVID-19 in Iranian workplaces. Work. 2021;69(2):327–30. doi: 10.3233/WOR-205045 34120923

[3] Zhang W-R, Wang K, Yin L, Zhao W-F, Xue Q, Peng M, et al. Mental health and psychosocial problems of medical health workers during the COVID-19 epidemic in China. Psychother Psychosom. 2020;89(4):242–50. doi: 10.1159/000507639 32272480 PMC7206349

[4] Catton H. Global challenges in health and health care for nurses and midwives everywhere. Int Nurs Rev. 2020;67(1):4–6. doi: 10.1111/inr.12578 32083728 PMC7165846

[5] Elhadi M, Msherghi A, Elgzairi M, Alhashimi A, Bouhuwaish A, Biala M, et al. Psychological status of healthcare workers during the civil war and COVID-19 pandemic: a cross-sectional study. J Psychosom Res. 2020;137:110221. doi: 10.1016/j.jpsychores.2020.110221 32827801 PMC7428743

[6] Organization WH. Coronavirus disease (COVID-19) outbreak: rights, roles and responsibilities of health workers, including key considerations for occupational safety and health: interim guidance, 19 March 2020. World Health Organization; 2020.

[7] Lai J, Ma S, Wang Y, Cai Z, Hu J, Wei N, et al. Factors associated with mental health outcomes among health care workers exposed to coronavirus disease 2019. JAMA Netw Open. 2020;3(3):e203976. doi: 10.1001/jamanetworkopen.2020.3976 32202646 PMC7090843

[8] Zamanzadeh V, Valizadeh L, Khajehgoodari M, Bagheriyeh F. Nurses’ experiences during the COVID-19 pandemic in Iran: a qualitative study. BMC Nurs. 2021;20:198. doi: 10.1186/s12912-021-00722-z 34649547 PMC8515778

[9] Organization WH. WHO calls for healthy, safe and decent working conditions for all health workers, amidst COVID-19 pandemic. World Day for Safety and Health at Work: WHO key facts & key messages to support the day. 2020.

[10] Lotfollahzadeh A, Rastgoo L, Shirinzadeh I, Kharghani Moghadam SM, Ebrahimi H. Investigating the compliance of COVID-19 protocols in the workplaces of Ardabil, Iran. Work. 2021;70(4):1031–7. doi: 10.3233/WOR-210551 34842217

[11] Diener E, Suh EM, Lucas RE, Smith HL. Subjective well-being: three decades of progress. Psychol Bull. 1999;125(2):276–302. doi: 10.1037/0033-2909.125.2.276

[12] Walton RE. Quality of working life: what is it. Sloan Manag Rev. 1973;15(1):11–21.

[13] Maslach CS. Job burnout. Ann Rev Psychol. 2001;Annual. 10.1146/annurev.psych.52.1.397 11148311

[14] Azizi L, Feyzabadi Z, Salehi M. Exploratory and confirmatory factor analysis of maslach burnout inventory among tehran universitys employees. 2008.

[15] Grande RAN, Butcon VER, Indonto MCL, Villacorte LM, Berdida DJE. Quality of life of nursing internship students in Saudi Arabia during the COVID-19 pandemic: a cross-sectional study. Int J Afr Nurs Sci. 2021;14:100301. doi: 10.1016/j.ijans.2021.100301 33824852 PMC8015389

[16] Mohammadi M, Mozaffari N, Dadkhah B, Etebari Asl F, Etebari Asl M. Study of work-related quality of life of nurses in Ardabil province hospitals. J Health Care. 2017;19(3):108–16.

[17] Griffin RW, Phillips JM, Gully SM. Organizational behavior: managing people and organizations. CENGAGE Learning; 2020.

[18] Moalemi S, Kavosi Z, Beygi N, Deghan A, Karimi A, Parvizi MM. Evaluation of the persian version of maslach burnout inventory-human services survey among iranian nurses: validity and reliability. Galen Med J. 2018;7:e995. doi: 10.22086/gmj.v0i0.995 34466422 PMC8343696

[19] Mokhtari R, Jaras M. Investigating the relationship between the quality of work life with turnover intention among nurses of Arak University of Medical Sciences hospitals in Corona pandemic, Iran. 2021.

[20] Mohamadzadeh Tabrizi Z, Mohammadzadeh F, Davarinia Motlagh Quchan A, Bahri N. COVID-19 anxiety and quality of life among Iranian nurses. BMC Nurs. 2022;21(1):27. doi: 10.1186/s12912-021-00800-2 35057763 PMC8771181

[21] Murat M, Köse S, Savaşer S. Determination of stress, depression and burnout levels of front-line nurses during the COVID-19 pandemic. Int J Ment Health Nurs. 2021;30(2):533–43. doi: 10.1111/inm.12818 33222350 PMC7753629

[22] Suryavanshi N, Kadam A, Dhumal G, Nimkar S, Mave V, Gupta A, et al. Mental health and quality of life among healthcare professionals during the COVID-19 pandemic in India. Brain Behav. 2020;10(11):e01837. doi: 10.1002/brb3.1837 32918403 PMC7667343

[23] Zheng R, Zhou Y, Fu Y, Xiang Q, Cheng F, Chen H, et al. Prevalence and associated factors of depression and anxiety among nurses during the outbreak of COVID-19 in China: a cross-sectional study. Int J Nurs Stud. 2021;114:103809. doi: 10.1016/j.ijnurstu.2020.103809 33207297 PMC7583612

[24] Labrague LJ, de Los Santos JAA. Fear of COVID-19, psychological distress, work satisfaction and turnover intention among frontline nurses. J Nurs Manag. 2021;29(3):395–403. doi: 10.1111/jonm.13168 32985046 PMC7537256

[25] Maslakçı A, Sürücü L, Sesen H. Fear of COVID-19 and work-quality of life among nurses: the mediating role of psychological well-being. Manag Sci Lett. 2021;11(7):1985–90.

[26] World Health Organization. COVID-19: Occupational health and safety for health workers: interim guidance. World Health Organization. 2021.

[27] Sihvola S, Nurmeksela A, Mikkonen S, Peltokoski J, Kvist T. Resilience, job satisfaction, intentions to leave nursing and quality of care among nurses during the COVID-19 pandemic - a questionnaire study. BMC Health Serv Res. 2023;23(1):632. doi: 10.1186/s12913-023-09648-5 37316918 PMC10266309

[28] Evans DP. COVID-19 and violence: a research call to action. BMC Women’s Health. 2020;20(1):1–3. 33172466 10.1186/s12905-020-01115-1 PMC7653443

[29] Buselli R, Corsi M, Baldanzi S, Chiumiento M, Del Lupo E, Dell’Oste V, et al. Professional quality of life and mental health outcomes among health care workers exposed to Sars-Cov-2 (Covid-19). Int J Environ Res Public Health. 2020;17(17):6180. doi: 10.3390/ijerph17176180 32858810 PMC7504107

